# Voluntary activation of muscle in humans: does serotonergic neuromodulation matter?

**DOI:** 10.1113/JP282565

**Published:** 2022-08-01

**Authors:** Justin J. Kavanagh, Janet L. Taylor

**Affiliations:** ^1^ Neural Control of Movement laboratory Menzies Health Institute Queensland Griffith University Gold Coast Australia; ^2^ Centre for Human Performance, School of Medical and Health Sciences Edith Cowan University Perth Australia; ^3^ Neuroscience Research Australia Sydney Australia

**Keywords:** fatigue, motoneurones, neuromodulation, persistent inward current, serotonin

## Abstract

Ionotropic inputs to motoneurones have the capacity to depolarise and hyperpolarise the motoneurone, whereas neuromodulatory inputs control the state of excitability of the motoneurone. Intracellular recordings of motoneurones from *in vitro* and *in situ* animal preparations have provided extraordinary insight into the mechanisms that underpin how neuromodulators regulate neuronal excitability. However, far fewer studies have attempted to translate the findings from cellular and molecular studies into a human model. In this review, we focus on the role that serotonin (5‐HT) plays in muscle activation in humans. 5‐HT is a potent regulator of neuronal firing rates, which can influence the force that can be generated by muscles during voluntary contractions. We firstly outline structural and functional characteristics of the serotonergic system, and then describe how motoneurone discharge can be facilitated and suppressed depending on the 5‐HT receptor subtype that is activated. We then provide a narrative on how 5‐HT effects can influence voluntary activation during muscle contractions in humans, and detail how 5‐HT may be a mediator of exercise‐induced fatigue that arises from the central nervous system.

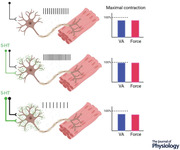

## Graphical abstract

**Abstract figure legend** Inputs to neuromodulatory receptors on motoneurones, such as those involved in the serotonergic system, modify the motoneurones’ responsiveness to ionotropic input. The release of serotonin (5‐HT) into the spinal cord is linked to the level of motor activity being performed, where 5‐HT can increase the discharge rate of motoneurones via excitatory 5‐HT receptors on the soma and dendrites. This in turn can lead to increased voluntary muscle activation (VA) and maximal force generation. However, intense release of 5‐HT onto motoneurones may lead to a spillover of 5‐HT into extracellular compartments to activate inhibitory 5‐HT receptors on the axon initial segment. This can cause a reduction in motoneurone discharge rate, thus decreasing VA and maximal force generation. To gain insight into the serotonergic contributions to muscle activation in humans, pharmacological interventions have been employed to enhance the concentration of 5‐HT in the central nervous system or activate selective 5‐HT receptors.

## Introduction

The final common pathway in the motor system is the motor unit, which consists of the α‐motoneurone in the spinal cord and all muscle fibres that it innervates. Given that the motor unit links the central nervous system to skeletal muscle, efficient and purposeful movement can only be achieved by tightly controlled motoneurone activity. The firing pattern of a motoneurone is produced by converting synaptic inputs into action potentials, where the regulation of input to output is determined by the intrinsic electrical properties of motoneurones (Kernell, 2006). Hence, to gain a better understanding of how the nervous system regulates motor activity, it is of great importance to explore how inputs to the motoneurone influence activation of muscle.

Synaptic inputs to motoneurones will typically activate two classes of receptors: ionotropic and neuromodulatory. In a general sense, inputs to ionotropic receptors are command signals from descending motor pathways, peripheral afferents, or spinal interneurons, which have the capacity to depolarise and hyperpolarise the motoneurone. Typically, ionotropic receptors are ligand‐gated ion channels which directly gate ion flow into cells to generate either an excitatory or inhibitory response in postsynaptic neurons (Alexander et al., 2011; Eccles & McGeer, 1979; Heckman & Binder, 1991; Powers et al., 2002). In contrast, inputs to neuromodulatory receptors control the state of excitability of the motoneurone through intracellular signalling pathways that modify the motoneurone's responsiveness to ionotropic input (Eccles & McGeer, 1979; Elliott & Wallis, 1992; Fedirchuk & Dai, 2004; Heckman, Hyngstrom et al., 2008; Murray et al., 2011). The role that neuromodulators play in activating motoneurones has received considerable attention in the past 30 years. Most notably, intracellular recordings of motoneurones from *in vitro* and *in situ* animal preparations have provided considerable insight into how electrical properties of motoneurones are influenced by neuromodulators. However, fewer studies have attempted to translate findings from intracellular experiments into humans, as there are considerable challenges in reproducing reductionist experiments in an intact human neuromuscular system.

There are several neuromodulator systems that regulate the excitability of cortical and spinal motor circuits, and many excellent reviews have been written that describe the effects of each system on motor activity (e.g. the cholinergic system: Deffains & Bergman, 2015; Jones, 2008; Mille et al., 2021; Naicker et al., 2017; the dopaminergic system: Arber & Costa, 2022; Ikeda et al., 2015; Klaus et al., 2019; Sharples et al., 2014; and the noradrenergic system: Balaban, 2002; Benarroch, 2018; Fung et al., 1994). Our Topical Review will provide a unique summary regarding how serotonergic neuromodulation contributes to voluntary activation of muscle in humans. To achieve this, the functional anatomy of serotonergic pathways with regard to the motor system will be outlined before summarising human experiments that assess the role of this neuromodulator in performing unfatigued and fatiguing muscle contractions.

### The serotonergic system

Serotonin (or 5‐hydroxytryptamine, 5‐HT) is a monoamine neurotransmitter that modulates the intrinsic properties of neurons in the central nervous system (CNS). In the periphery, it is synthesised in enteric mucosal cells, influences gut motility among other functions, and is stored in platelets (Terry & Margolis, 2017). However, 5‐HT is unable to cross the blood–brain barrier and must be synthesised within the CNS from its precursor, tryptophan. Hence, the primary site of CNS 5‐HT synthesis in vertebrates is the raphe nuclei of the brainstem (Hery et al., 1982; Hornung, 2003; Imai et al., 1986; Pollak Dorocic et al., 2014). At a cellular level, 5‐HT is released into synapses and exerts pre‐ and postsynaptic effects when it binds to membrane receptors. It is often suggested that the termination of 5‐HT's effects at the synapse occurs with reuptake into presynaptic terminals or glial cells via monoamine transporters. However, serotonergic effects on neuronal function may not necessarily end with reuptake of 5‐HT, as activation of G‐protein‐coupled receptors can elicit long‐lasting changes in ion channel function (Pavlos & Friedman, 2017). Seven classes of 5‐HT receptors have been identified (Lucas & Hen, 1995). They are all G‐protein‐coupled receptors (Hoyer & Martin, 1997) except for the 5‐HT_3_, receptor which is a ligand‐activated channel (Thompson & Lummis, 2006). However, the principal 5‐HT receptors expressed by motoneurones are 5‐HT_1A_, 5‐HT_1B_, 5‐HT_2A_, 5‐HT_2B_ and 5‐HT_2C_. (For comprehensive reviews on 5‐HT receptor subtypes in the CNS refer to Barnes & Sharp, 1999; Nichols & Nichols, 2008; Peroutka, 1990; Perrier et al., 2013.)

**Figure 1:**
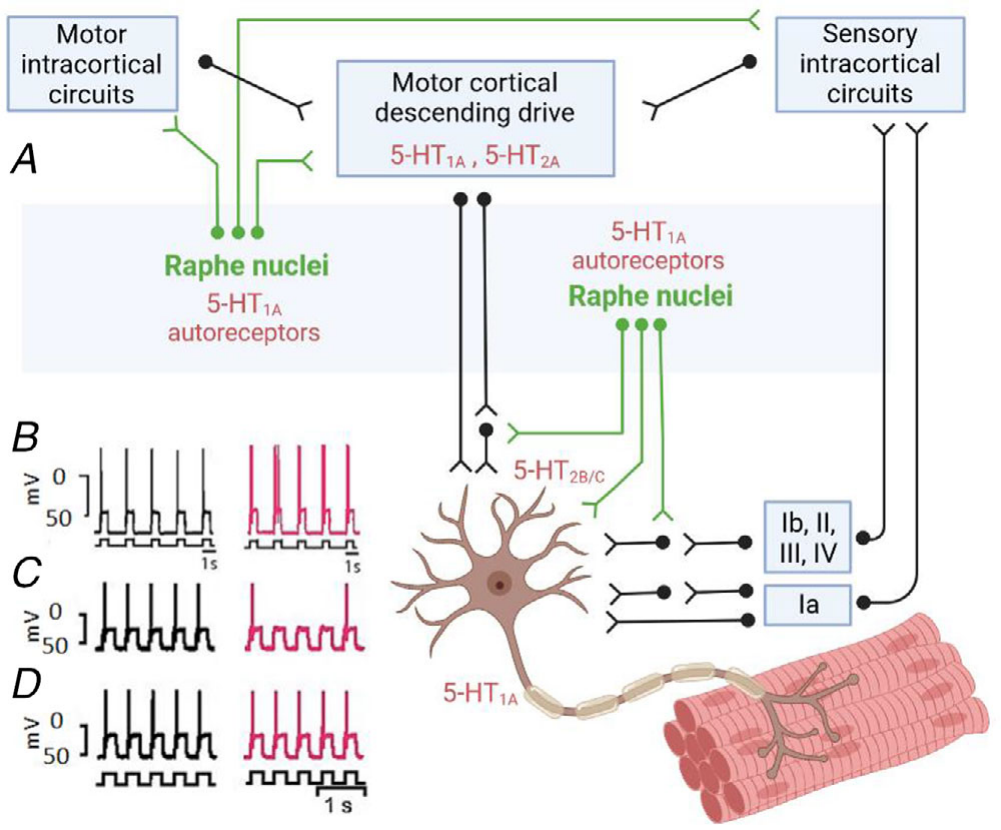
Serotonergic activity modulates cortical and spinal motoneurone activity *A*, serotonin synthesis in the mammalian CNS occurs in the raphe complex of the brainstem. Rostral raphe nuclei ascend primarily to the forebrain to innervate virtually all regions. Caudal raphe nuclei project mainly to the cerebellum and spinal cord, where the monosynaptic raphe–spinal pathway releases 5‐HT on the soma and dendrites of spinal motoneurones. Ionotropic inputs from descending motor cortical pathways, peripheral afferents, or spinal interneurons, have the capacity to depolarise and hyperpolarise the motoneurone. However, activation of 5‐HT receptors, predominantly in the 5‐HT_1_ and 5‐HT_2_ families, regulates the state of excitability of the motoneurone via intracellular signalling pathways to modify the motoneurone's responsiveness to ionotropic input. *B*, intracellular recordings from motoneurones in an adult turtle spinal cord preparation (black). The number of action potentials evoked by depolarizing current pulses injected in the soma is increased following 1 s of 40 Hz electrical stimulation of the dorsolateral funiculus (red), which contains descending serotonergic fibres of the raphe–spinal pathway. *C*, lengthening the duration to 3 min of 40 Hz electrical stimulation inhibited motoneurone firing compared to the control, which is attributed to 5‐HT spillover onto extracellular 5‐HT_1A_ receptors on the axon initial segment. *D*, 3 min of 40 Hz stimulation in the presence of the 5‐HT_1A_ antagonist WAY‐100635 (20 μM) removed the inhibitory influence of 5‐HT. Data sourced from Cotel et al. (2013).

From the brainstem, neurons from a group of serotonergic nuclei *ascend* to distribute across many areas of the brain, including the frontal lobe where 5‐HT can be released in the cerebral cortex (Fig. 1*A*). A high density of 5‐HT fibres has been identified in motor areas of the rat and primate cortex (Vertes, 1991; Wilson & Molliver, 1991), but the role of these rostral raphe projections in motor activity has not been well‐defined. Cortical structures predominantly express receptors from the inhibitory 5‐HT_1_ family and to a lesser extent the excitatory 5‐HT_2_ family (Celada et al., 2013; Pazos & Palacios, 1985; Pazos et al., 1985), where 5‐HT_1A_ is expressed extensively in laminae II/III of the motor cortex and 5‐HT_2A_ receptors are uniformly distributed across motor cortex laminae (Joyce et al., 1993; Vitrac & Benoit‐Marand, 2017). It appears that depleting brain 5‐HT in rats has the effect of reducing the excitability of neural circuits in motor cortex (Scullion et al., 2013), and administration of selective serotonin reuptake inhibitors (SSRIs) enhances excitability in motor cortex circuits in humans (Gerdelat‐Mas et al., 2005; Ilic et al., 2002). Thus, a likely key role of 5‐HT in the motor cortex is modulation of facilitatory intracortical motor circuits. 5‐HT also appears to play a critical role in plasticity of motor circuits, as SSRI administration combined with either non‐invasive brain stimulation or motor practice can influence excitatory synaptic plasticity. In particular, there is evidence to suggest enhanced long‐term potentiation‐like plasticity from paired associative stimulation (Batsikadze et al., 2013; Nitsche et al., 2009), whereas changes in plasticity from practicing motor tasks has been observed in most (Classen et al., 1998; Loubinoux et al., 2002; Pleger et al., 2004), but not all (McDonnell et al., 2018), experiments that have assessed cortical plasticity after SSRI ingestion. Functional magnetic resonance imaging has identified several interconnected structures that are affected by enhanced 5‐HT concentration, where short‐term drug‐induced neuroplastic changes are associated with enhanced activation of primary motor and premotor cortices, as well as posterior supplementary motor areas (i.e. executive motor areas of the cortex) (Loubinoux et al., 1999).

Most of the work that has examined 5‐HT effects on motor cortical activity has employed magnetic or electrical stimulation techniques to explore cortical plasticity, or neuroimaging techniques to map changes in regional activity due to changes in neurotransmitter concentration. These approaches provide immense insight to the cortex itself, but do not necessarily link 5‐HT changes in the CNS to motor performance. Instead, considerably more is known about how 5‐HT release on motor circuits in the spinal cord directly affects motor activity. From the brainstem, the raphe–spinal pathway *descends* to the spinal cord to form well‐defined synapses with motoneurones, afferent neurons, and interneurons (Fig. 1*A*) (Alvarez et al., 1998; Kawashima, 2018; Ridet et al., 1994). 5‐HT release into the dorsal horn and intermediate zone of the spinal cord can cause remarkably complex outcomes at the neuronal level, as 5‐HT can either depress or facilitate transmission in afferent fibres (Belcher et al., 1978; Jordan et al., 1979; Todd & Millar, 1983), and the response to 5‐HT can differ depending on the threshold for afferent activation (Belcher et al., 1978; Jankowska et al., 1993). Adding further complexity to our understanding of how 5‐HT regulates sensory neurones in the spinal cord is that 5‐HT's effects may be exerted presynaptically and postsynaptically. In particular, modulation by 5‐HT of the actions of muscle spindle (Ia fibres) and tendon organ (II fibres) afferents on spinal interneurons depends on the type of afferent that is activated and the functional type of the interneuron (Jankowska et al., 2000). Thus, it is difficult to predict how motor function may be influenced by the actions of 5‐HT on afferent neurons and interneurons. Instead, the most direct effects of 5‐HT on motor function are via the monosynaptic connections to the motoneurones from the fibres in the raphe–spinal pathway to the motoneurones. There are approximately 1500 serotoninergic contacts on each motoneurone (Alvarez et al., 1998), where the release of 5‐HT occurs 0.5–1.0 s following the onset of detectable neural activation of locomotor muscles (Noga et al., 2017). The intensity of 5‐HT release from the raphe‐spinal pathway, but not the ascending pathway, is thought to correspond to the intensity of motor activity being performed by upper limb (forelimb) and lower limb (hindlimb) muscles. This viewpoint has predominantly been based on cat studies that show activity in the raphe nuclei is abolished during sleep (Cespuglio et al., 1981; McGinty & Harper, 1976), but near‐linearly scaled to locomotion speed (Jacobs & Fornal, 1997; Jacobs et al., 2002; Veasey et al., 1995). Incremental increases in treadmill walking speed correspond to incremental increases in the discharge rate of single raphe‐spinal fibres (Veasey et al., 1995), which potentially points towards a contraction intensity dependent mechanism of 5‐HT release. On face value, this suggests a very useful motor control strategy, in which strong contractions cause large release of 5‐HT to amplify signals from synaptic inputs. However, a phenomenon exists where the presence of too much 5‐HT in the CNS can limit the ability of motoneurons to activate muscle.

### Dual role of 5‐HT on motoneurone activity

There is widespread agreement that 5‐HT promotes depolarisation in motoneurones, which arises from several concomitant mechanisms. In particular, cellular and molecular experiments have identified that 5‐HT can facilitate a rectifying inward current (Hsiao et al., 1997; Takahashi & Berger, 1990), facilitate low threshold Ca^+^ currents (Berger & Takahashi, 1990), or inhibit K^+^ leak conductance (Elliott & Wallis, 1992; Perrier et al., 2003) to increase neuronal excitability. 5‐HT can also increase discharge rate via mechanisms that reduce the amplitude of slow and medium afterhyperpolarisation phases that follow the action potential (Bayliss et al., 1995; Grunnet et al., 2004; Hounsgaard & Kiehn, 1989; Hounsgaard et al., 1988; Wikstrom et al., 1995). Although 5‐HT can reduce hyperpolarisation by decreasing the threshold for generating Na^+^‐based action potentials (Fedirchuk & Dai, 2004), 5‐HT also generates a Ca^+^‐dependent plateau potential by reducing a K^+^ current responsible for slow afterhyperpolarisation (Hounsgaard & Kiehn, 1989). In general, the functional role of 5‐HT on motoneurone activity has been revealed by slice preparations, partially intact animal preparations, and simulations. With these approaches, it has been possible to examine the interaction between physiological 5‐HT concentrations and 5‐HT receptor subtypes. Electrical stimulation of the dorsolateral funiculus (DLF; contains descending serotonergic fibres) in integrated preparations of the adult turtle spinal cord has been shown to promote the release of endogenous 5‐HT into the ventral horn of the spinal cord (Delgado‐Lezama et al., 1997; Perrier & Cotel, 2008b; Perrier & Delgado‐Lezama, 2005). In this circumstance, motoneurone recordings have confirmed that discharge rate increases with brief bursts of 5‐HT release (Fig. 1*B*) (Hounsgaard & Kiehn, 1989; Perrier & Delgado‐Lezama, 2005; Perrier & Hounsgaard, 2003), and this increase is mediated by activation of 5‐HT_2B/C_ receptors located on the dendrites and the cell body of the motoneurone (Cotel et al., 2013; Jackson & White, 1990).

In addition to 5‐HT exerting excitatory effects on motoneurones, the presence of this neuromodulator has also been observed to create an inhibitory effect on motor activity. Early *in vivo* work that recorded motoneurone activity in cats observed that 5‐HT injected in the vicinity of the motoneurone caused hyperpolarisation of the resting membrane, which led to a failure of the motoneurone to fire (Phillis et al., 1968). An underlying mechanism of 5‐HT‐mediated inhibition of motoneurone activity was clarified in a series of elegant experiments by Cotel and Perrier. Using preparations from the turtle spinal cord, it was revealed that the ability of motoneurones to fire is inhibited when 5‐HT is iontophoretically applied close to motoneurone soma (Perrier & Hounsgaard, 2003) and when prolonged DLF stimulation causes large endogenous release of 5‐HT onto motoneurones (Fig. 1*C*) (Cotel et al., 2013; Perrier et al., 2017). The mechanism underlying 5‐HT's dual excitatory and inhibitory effects aligns with the compartmentalisation of 5‐HT receptor subtypes expressed on motoneurones. The monosynaptic connections that raphe‐spinal neurons have with motoneurones causes direct 5‐HT release onto receptors in somatodendritic regions to enhance motoneurone excitability. However, activation of 5‐HT_1A_ receptors expressed in perisomatic regions inhibits Na^+^ channels responsible for the genesis of action potentials, which has the effect of preventing motoneurone firing (Perrier & Cotel, 2008a). Given that the inhibitory 5‐HT_1A_ receptors are only located on the axon initial segment, and the axon initial segment is devoid of serotonergic innervation, activation of 5‐HT_1A_ receptors can only occur if extracellular concentrations of 5‐HT are large enough to spill over onto the axon initial segment (Cotel et al., 2013; Perrier & Cotel, 2015; Perrier et al., 2017). These findings provide a potential cellular mechanism for central fatigue and provide a foundation to investigate if 5‐HT activity during prolonged bouts of physical activity reduces motor output in humans. However, it is worth noting that these findings are only reported for adult turtle motoneurones and may not be applicable for other species. Indeed, a recent study has identified 5‐HT boutons present on the axon initial segment of rodent motoneurones (Deardorff et al., 2021), and compartmentalisation of 5‐HT receptor subtypes on human motoneurones is yet to be detailed. Finally, other activity‐dependent mechanisms may be influenced by serotonin. For example, sodium–potassium pump activity can cause inhibitory effects on motoneurone discharge during rhythmic activity (Zhang & Sillar, 2012; Zhang et al., 2015; Picton & Sillar, 2016). In tadpoles, this effect can be modulated bidirectionally through 5‐HT_2A_ and 5‐HT_7_ receptors (Hachoumi et al., 2022), although this has not yet been demonstrated in mammals.

### Is serotonergic neuromodulation reflected in voluntary muscle activation in humans

**Figure 2:**
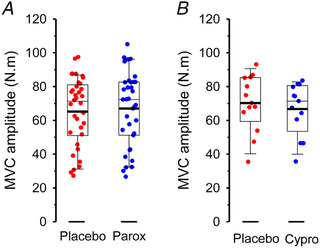
Elbow flexion MVC torque after ingestion of paroxetine or cyproheptadine *A*, the SSRI paroxetine (Parox) exerts small, but significant, increases in torque during brief unfatigued MVCs (paired Student's *t*‐test, *P* = 0.046). Given that voluntary activation of biceps is high in healthy individuals (e.g. 97–99%), a ceiling effect exists whereby only small increases in voluntary activation and elbow flexion torque are possible. *B*, competitive antagonism of the 5‐HT_2_ receptor via cyproheptadine (Cypro) causes significant reductions in the ability to generate elbow flexion torque during brief unfatigued MVCs (paired *t*‐test, *P* = 0.003). Data are sourced from a combination of studies from the host laboratory using healthy adults aged 20–30 year (Henderson et al., 2022; Kavanagh et al., 2019; Thorstensen et al., 2020; Thorstensen et al., 2021).

Although it is difficult to identify how individual neuromodulators contribute to motor activity, simulations indicate that the net effect of maximal neuromodulation may be a three‐ to five‐fold amplification of the currents that the motoneurons receive from synaptic inputs (Hultborn et al., 2003; Lee & Heckman, 2000). Hence, the availability of 5‐HT is critical to enhancing the gain of motoneurone output, and ultimately, activating the muscle to produce force. Data from healthy adults performing maximal effort contractions mostly support this finding. However, the magnitude of response in human experiments is small (Fig. 2*A* and *B*). Following the administration of the SSRI paroxetine to increase serotonin availability, the force of maximal voluntary contractions (MVC) has been noted to increase 1.5–4.5% in several experiments employing elbow flexion protocols. Similarly, voluntary activation increased by 0.3% when measured using cortical stimulation (Thorstensen et al., 2020) and by ∼1.3% when measured using motor nerve stimulation (Kavanagh et al., 2019; Thorstensen et al., 2020). A finding of only small drug‐related increases in voluntary activation is perhaps not surprising, as increases in motoneurone excitation during near‐maximal contraction intensities produce only small changes in interpolated twitch amplitude (Herbert & Gandevia, 1999). Hence, voluntary activation of the biceps (which is calculated from interpolated twitches) may be as high as 98% or 99% in healthy individuals, and any intervention that may increase activation is limited by a very close ceiling (i.e. 100% voluntary activation). Nevertheless, an enhanced ability to activate the muscle is consistent with 5‐HT effects that are mediated by excitatory somato‐dendritic 5‐HT_2_ receptors on motoneurones. Consistent with this, the administration of a 5‐HT_2_ competitive antagonist leads to the opposite effect on maximal force generation. Maximal elbow flexion force declined by ∼3% after the administration of cyproheptadine (Thorstensen et al., 2021). Antagonism of the 5‐HT_2_ receptor also leads to a reduction in rate of force development when performing rapid dorsiflexions, which is mediated by reduced firing rates in motor units of the tibialis anterior (Goodlich et al., 2022). Thus, enhancing 5‐HT availability during strong unfatigued contractions increases force generation, whereas blocking 5‐HT_2_ receptor activity decreases force generation via neural mechanisms in healthy individuals.

Persistent inward currents (PIC) play a critical role in setting the gain of motoneurones. PICs are mediated by voltage‐gated sodium channels (Na_v_1.1 and Na_v_1.6) and L‐type voltage‐gated calcium channels (Ca_v_1.2 and Ca_v_1.3) on somato‐dendritic surfaces of motoneurones (Heckmann et al., 2005; Schwindt & Crill, 1977; Schwindt & Crill, 1980). In mammals, there are believed to be equal contributions from slow activating L‐type Ca^2+^ current and a fast activating persistent Na^+^ current to the PIC (Heckman, Hyngstrom et al., 2008). However, recent evidence shows that non‐linearities in motoneurone discharge may have more complex mechanisms including activation of TRPm5 and inactivation of Kv1.2 channels (Bos et al., 2018; Bos et al., 2021). Nonetheless, PICs are strongly influenced by monoamines. Activation of PICs causes a remarkable amplification of depolarising drive to the motoneurone and evokes a strong acceleration of motoneurone firing rate (Bennett et al., 1998; Hounsgaard et al., 1988; Lee & Heckman, 1998). Thus, it might be expected that PICs may be partially responsible for 5‐HT‐mediated increases in voluntary activation during MVCs. In humans, the amplitude of PIC activation can only be estimated. The accepted technique for this estimation for an individual motoneurone compares the estimated synaptic input that recruits a motoneurone to that at its derecruitment (i.e. the motoneurone's hysteresis). PICs provide little drive to the motoneurone at recruitment as initiation of an action potential and the activation of PICs occur at a similar membrane potential (but see Afsharipour et al., 2020 for important nuances with regard to variations in PIC threshold). By comparison, PICs are fully active after the motoneurone has been firing for ∼0.5–1.0 s (Heckman, Johnson et al., 2008; Lee & Heckman, 1996). Thus, at derecruitment, PICs contribute part of the depolarising current for motoneurone firing so that less synaptic input is required.

The paired motor unit technique uses the firing rate of low threshold motor units, which already have PICs fully active, as estimates of synaptic input to other motor units in the same pool. The measure that is calculated is the difference in frequency of firing of the lower threshold motor unit at recruitment and derecruitment of the higher threshold motor unit (known as ∆*F*). Discharge rates are recorded from intramuscular electromyography electrodes (Foley & Kalmar, 2019; Gorassini et al., 2002; Udina et al., 2010) or extracted from high‐density electromyography arrays placed over the muscle (Afsharipour et al., 2020; Hassan et al., 2020). The idea that the influence of serotonin on human motor performance is mediated via PICs is supported by pharmacological studies in a small number of healthy participants (*n* = 3), in whom ∆*F* increased with ingestion of an SSRI or decreased with a 5‐HT_2_ receptor antagonist (D'Amico et al., 2013). Because ∆*F* has only been calculated during submaximal ramp‐shaped contractions, it is unknown if PICs are directly linked to enhanced MVCs in humans. However, it is known that PICs associated with gastrocnemius medialis increase from 10% to 20% MVC and PICs associated with soleus increase from 10% to 30% MVC during slow ramped plantarflexions (Orssatto, Mackay et al., 2021). These findings provide support that PIC activation in humans is linked to voluntary drive and hence 5‐HT release. The difference between gastrocnemius and soleus also highlights that PICs, and their influence on motoneuron intrinsic properties, differ between muscles and motoneuron subtypes (Huh et al., 2017; Lee & Heckman, 1998). Reductions in motoneurone PIC may also provide a mechanism for loss of force production from skeletal muscle. In younger populations, there is evidence that ∆*F* reduces up to 25% for soleus motoneurones following passive stretching of the plantarflexors, which may explain some of the reductions in force associated with stretching protocols (Trajano et al., 2020). In older populations, ∆*F* is lower than in young adults for soleus and tibialis anterior (Orssatto, Borg et al., 2021), and biceps and triceps brachii (Hassan et al., 2021), and may contribute to age‐related reductions in motoneurone firing rates and muscle force. However, the mechanisms underlying the reductions in PIC activity are not known. Conceivably, monoamine release onto the motoneurones could be reduced or the motoneuronal response to monoamines could be altered by changes in receptors. Alternatively, PICs are sensitive to inhibitory synaptic input and could be turned off by added inhibition.

### Is prolonged release of serotonin a mechanism of fatigue in humans?

Serotonin has long been implicated in the development of fatigue in humans, where the performance of prolonged exercise increases blood tryptophan levels and thus 5‐HT synthesis in the CNS (Newsholme et al., 1987). The ‘central fatigue hypothesis’ was introduced in the 1980s, when it was proposed that enhanced concentration of brain 5‐HT induced negative effects on arousal and mood, and increased lethargy and sleepiness. It was postulated that this mechanism could influence the perception of effort and, therefore, fatigue (Newsholme & Blomstrand, 2006; Newsholme et al., 1987). Following this original hypothesis, several studies used pharmacological interventions to modify CNS 5‐HT levels during prolonged cycling, and found that cycling performance was sometimes (Meeusen et al., 2001; Roelands et al., 2009; Strachan et al., 2004), but not always (Strüder et al., 1998; Teixeira‐Coelho et al., 2014; Wilson & Maughan, 1992), limited by enhanced 5‐HT concentrations. These mixed findings are most likely a reflection on the complexity of neurotransmitter systems, and no single neurotransmitter is responsible for exercise‐induced central fatigue. Indeed, pharmacological interventions that antagonise D2 dopaminergic receptors (Thorstensen et al., 2018) and inhibit noradrenaline reuptake (Klass et al., 2012; Klass et al., 2016) have been shown to reduce voluntary muscle activation during fatiguing contractions in humans. Another difficulty with interpretation of pharmacological interventions during whole‐body exercise is that widespread, and often contradictory, interactions of serotonin occur for the motor system. Even at a supraspinal level, if increased cortical 5‐HT does reduce motivation and increase effort through actions at high level motor areas, increased motor cortex excitability with SSRI ingestion suggests that corticospinal pathways may be facilitated (Gerdelat‐Mas et al., 2005; Ilic et al., 2002).

**Figure 3:**
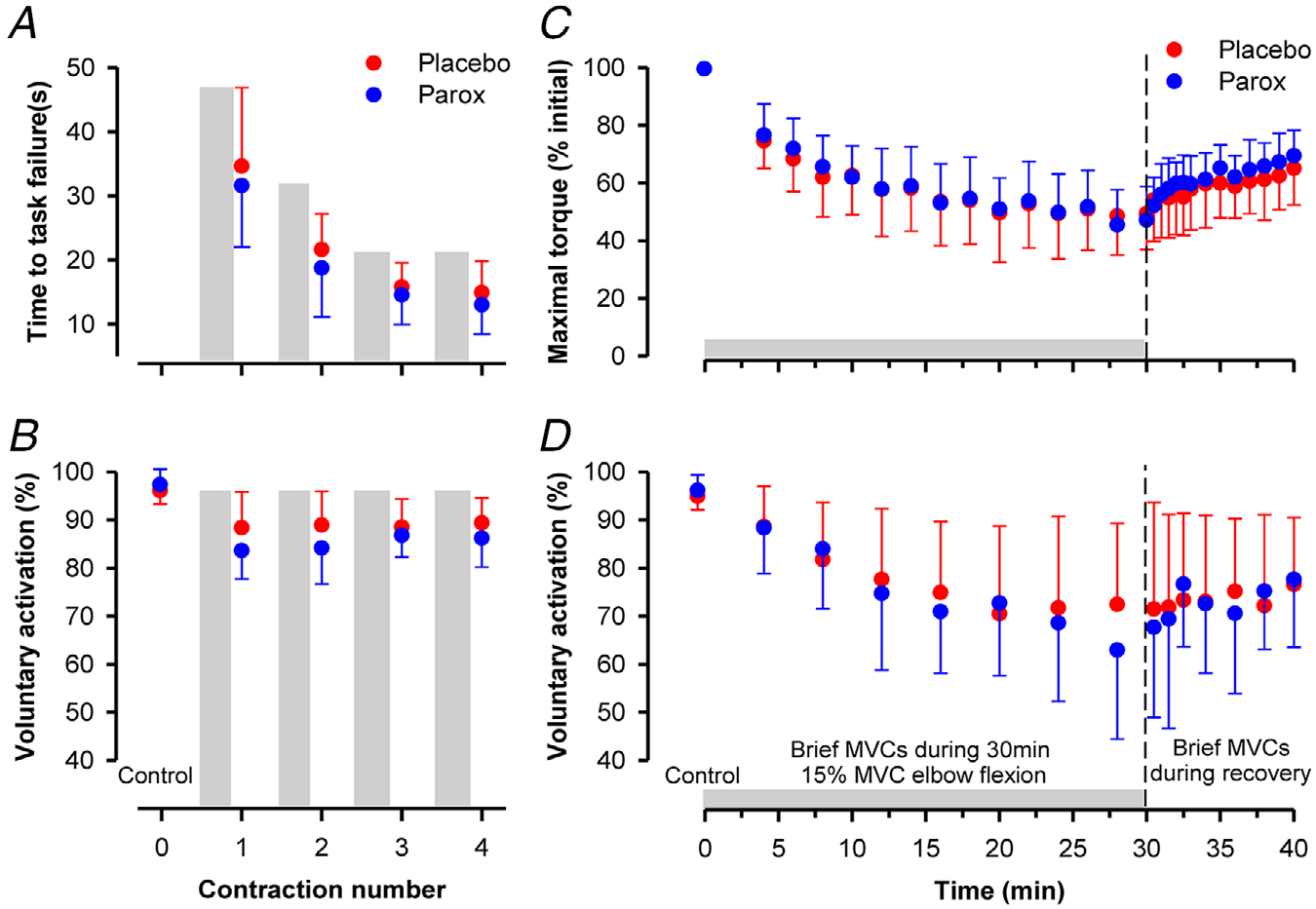
The effect of enhanced availability of 5‐HT on maximal and submaximal fatiguing isometric contractions Paroxetine (Parox) was used to enhance 5‐HT concentrations by reuptake inhibition. Motor nerve stimulation was used to assess voluntary activation of the elbow flexors. *A*, time‐to‐task failure was assessed for four sustained maximal elbow flexions, where each contraction was maintained until force declined to 60% MVC. Grey bars indicate a fatiguing contraction has occurred. A rest period of 40 s occurred between maximal contractions. *B*, each fatiguing contraction was followed 3 s later by a resting twitch, and then 3 s later by a brief maximal contraction with superimposed twitch, where voluntary activation was calculated as: [1 − (superimposed twitch/resting twitch)] × 100. Enhanced 5‐HT concentration caused a significantly greater fatigue‐related reduction in time‐to‐task failure (*P* = 0.038) and voluntary activation (*P* = 0.048). *C*, elbow flexion torque has also been assessed during a sustained 15% MVC, where the contraction was held for 30 min and fatigue responses continued to be monitored for 10 min of recovery. *D*, superimposed and resting twitches were obtained periodically throughout the contraction protocol and during recovery to calculate voluntary activation. There were no changes to fatigue‐related reductions in MVC torque (*P* = 0.376) and voluntary activation (*P* = 0.505) due to enhanced 5‐HT availability. Data sourced from Kavanagh et al. (2019) and Thorstensen et al. (2020).

Benchtop experiments cannot replicate the same physiological conditions associated with exercise‐induced fatigue. However, recordings obtained from animal preparations strongly suggest that activation of 5‐HT_1A_ receptors in the spinal cord would limit the ability for the CNS to activate muscle, and thus contribute to central fatigue. This specific mechanism appears relevant to humans, as ingestion of the 5‐HT_1A_ receptor agonist buspirone suppresses spinal motoneurone excitability (D'Amico et al., 2017) and reduces the capacity to perform prolonged bouts of exercise in healthy individuals (Marvin et al., 1997). However, exogenous activation of the 5‐HT_1A_ receptor may cause different responses to naturally occurring 5‐HT dynamics, so we performed several experiments to assess muscle activation when endogenously released 5‐HT is accumulated in the CNS. Fatiguing prolonged repeated *maximal* effort contractions were performed with and without enhanced availability of 5‐HT caused by ingestion of an SSRI. We observed that MVC torque and time‐to‐task failure were reduced when more 5‐HT was available (Fig. 3*A*) (Kavanagh et al., 2019). That is, more fatigue developed more quickly. It was evident that this decline in performance had neural origins, as fatigue‐related failure of voluntary activation was greater in the presence of higher 5‐HT concentrations (Fig. 3*B*). Additional experiments were performed using the hand muscle abductor digiti minimi, where fatigue‐related reductions in F‐wave persistence and F‐wave area following a prolonged MVC were substantially greater with greater 5‐HT availability. As F waves are a marker of motoneurone excitability, these findings indicated that reduced excitability of spinal motoneurones contributed to the added central fatigue caused by 5‐HT reuptake inhibition. Consistent with the idea of serotonin spillover as described above, the SSRI‐related accumulation of 5‐HT in the spinal cord could be due to strong serotonergic drive occurring when neural drive to the target muscle was high. The high levels of synaptic serotonin could then spill over to activate the extra‐synaptic inhibitory 5‐HT_1A_ receptors on motoneurones to suppress voluntary muscle activation.

Prolonged low‐intensity contractions can also lead to substantial declines in motor performance due to central fatigue (Sogaard et al., 2006). During the performance of a 15% MVC for 30 min, the development of muscle fatigue was demonstrated by a reduction in force of occasional brief MVCs and central fatigue by associated declines in voluntary activation. By comparing performance with and without serotonin reuptake inhibition, we were able to test if 5‐HT effects progressively limit muscle activation over time as the sustained contraction presumably causes a sustained release of 5‐HT in the CNS (Thorstensen et al., 2020). Contrary to our hypothesis, muscle force, voluntary activation and corticospinal excitability were not affected by 5‐HT reuptake inhibition during the prolonged low‐intensity contraction (Fig. 3*C* and *D*). The absence of 5‐HT effects may have occurred due to two possible mechanisms. First, during prolonged low‐intensity contraction, neural drive to the muscle may not have been of a sufficient intensity to cause intense release of 5‐HT from the raphe‐spinal pathway onto motoneurons. Hence, serotonin reuptake may have kept up with serotonin release despite the SSRI. Second, serotonergic drive may have declined throughout the contraction and caused a reduction of 5‐HT release onto motoneurones. The latter mechanism has support from cat experiments as the firing of raphe‐spinal neurons is reported to progressively decrease during sustained physical activity. In single raphe‐spinal neurons activity decreased by up to 50% after 40 min of treadmill walking (Fornal et al., 2006). Thus, serotonergic neuromodulation may not be an endless resource during muscle contractions. As yet, it is not clear what level and duration of voluntary contraction is enough to elicit fatigue‐related motoneurone inhibition through serotonin spillover. Our experiments suggest that 30 min of a sustained 15% MVC is insufficient, but 40–60 s of a sustained MVC is sufficient.

### Afferent feedback can potentially regulate 5‐HT effects at the motoneurone

Voluntary activation of muscle can be modulated by muscle afferent feedback. This presents a challenge for understanding motoneurone excitability, as ionotropic input received by the motoneuron comprises concurrent excitatory (descending drive and muscle spindle afferents) and inhibitory (group Ib, III and IV) synaptic activity that can be modulated by 5‐HT (Fig. 1). Adding to this complexity, most muscle afferents synapse on interneurons in the spinal cord to cause excitation and/or inhibition in multiple target muscles. Thus, examining the action of individual afferents, and recording activity from individual muscles, may not reflect the net output of the motor system (D'Amico et al., 2014).

Although the amplitude of dendritic PICs is directly proportional to the intensity of brainstem neuromodulatory drive, inhibition from low‐threshold sensory inputs has a strong suppressive effect on PIC amplitude. When inhibition is strong enough, the PICs created by the monoaminergic drive are reduced or even deactivated (Hultborn et al., 2003; Kuo et al., 2003). *In vivo* cat experiments have revealed that reciprocal inhibition may be especially critical for regulating PIC amplitude during functional motor activity. Even minor rotations of the ankle joint that cause barely detectable changes in reciprocal inhibition of motoneurones will reduce PICs by ∼50% (Hyngstrom et al., 2007). The likely candidate for this afferent mechanism is Ia disynaptic reciprocal inhibition evoked by length changes in the antagonist muscle, as other afferents are relatively insensitive to the changes in muscle length that occurred with passive rotation of the cat ankle in the experiment (Hyngstrom et al., 2007). The notion that reciprocal inhibition can dampen the effects of PICs has also been demonstrated in humans, where 128 Hz vibration of the tibialis anterior tendon to activate dorsiflexor muscle spindle afferents decreases Δ*F* for both the soleus and medial gastrocnemius during 30% MVC plantarflexions, and vibration of the Achilles tendon decreases Δ*F* for the tibialis anterior during 30% MVC dorsiflexions (Pearcey et al., 2020). Another human study found that 1 Hz electrical stimulation of the common peroneal nerve also has the capacity to reduce Δ*F* in medial gastrocnemius motor units in healthy individuals, which builds further support that Ia reciprocal inhibition reduces the contribution of PICs to MU firing in humans. (Mesquita et al., 2022). Interestingly, reciprocal inhibition may solve a motor control problem that arises from the diffuse projection of monoaminergic fibres in the spinal cord. Widespread projections from the serotonergic system will release 5‐HT on more than one motor pool during voluntary contractions. Hence, 5‐HT may simultaneously enhance PIC activity on multiple motoneurones, which has the potential to create a bias towards co‐contraction due to increasing excitability in antagonist motor pools (Heckman, Hyngstrom et al., 2008). A reasonable proposal suggests that Ia reciprocal inhibition may promote voluntary activation during a variety of motor tasks by only enhancing activity in task specific muscles from a background of diffuse excitatory neuromodulation (Heckman, Hyngstrom et al., 2008; Hyngstrom et al., 2007; Hyngstrom et al., 2008). Thus, it is possible that a 5‐HT mechanism involving inhibitory spinal circuitry can regulate the amplitude of agonist and antagonist muscle contractions.

### So does serotonergic neuromodulation matter in humans?

Animal experiments provide clear evidence that 5‐HT is a potent modulator of spinal circuits and motoneurone output. However, the effects of 5‐HT on voluntary muscle activity in humans are less clear. Effects on motor performance during whole‐body exercise are inconsistent. This is not to say that serotonergic neuromodulation does not matter for humans, but instead highlights the challenges associated with studying how a complex neuromodulatory system acts during muscle contractions. Controlled experiments using single‐joint, single muscle, contraction protocols have found that maximal force can be changed by altering 5‐HT activity in the CNS. The differences observed in voluntary activation are small but are present despite an otherwise intact system and the actions of other neuromodulators, including noradrenaline. This suggests that serotonin has a non‐redundant role in maximal voluntary contractions but still begs the question of exactly how important it is in typical motor tasks. Nonetheless, 5‐HT‐related changes in muscle activation typically emerge with strong contractions for both the unfatigued and fatigued motor system. Thus, it appears that the magnitude of descending drive to the muscle may be aligned with the level of 5‐HT neuromodulation in humans. Indeed, we are beginning to reveal evidence where 5‐HT's effects may be scaled to the intensity of muscle activation in humans (Goodlich et al., 2022; Henderson et al., 2022).

An inability to quantify 5‐HT release onto motoneurones, as well as quantifying the binding affinity to different 5‐HT receptor subtypes, presents a challenge for interpreting any neuromodulation experiment (not just human studies). Participant safety is an additional challenge in human experiments that use pharmacology to manipulate neurotransmitter activity. Human studies must operate within a window of safe drug administration, and typically use therapeutic doses of 5‐HT‐modulating medications. Thus, very little is known about how dosage effects influence 5‐HT activity in humans. Pharmacological interventions are also non‐specific, and will not have 100% effectiveness when competitively agonising, antagonising or inhibiting 5‐HT reuptake in humans. Individual variation in pharmacokinetics and drug responses also poses a challenge when studying humans, but variability in 5‐HT responses is also a prevalent feature of *in vitro* and *in vivo* animal experiments that examine motoneurone and afferent activity. It is our position that none of these factors should prevent human 5‐HT research from continuing, but instead should encourage scientists to further explore the relationship between neuromodulation and voluntary muscle activation.

## Additional information

### Competing interests

None.

### Author contributions

Both authors contributed to the conception and the design of this work, as well as the drafting and final approval of the manuscript. All authors have read and approved the final version of this manuscript and agree to be accountable for all aspects of the work in ensuring that questions related to the accuracy or integrity of any part of the work are appropriately investigated and resolved. All persons designated as authors qualify for authorship, and all those who qualify for authorship are listed.

### Funding

No funding was received for this work.

## Supporting information




Peer Review History

## References

[tjp15192-bib-0001] Afsharipour, B. , Manzur, N. , Duchcherer, J. , Fenrich, K. F. , Thompson, C. K. , Negro, F. , Quinlan, K. A. , Bennett, D. J. , & Gorassini, M. A. (2020). Estimation of self‐sustained activity produced by persistent inward currents using firing rate profiles of multiple motor units in humans. Journal of Neurophysiology, 124(1), 63–85. 32459555 10.1152/jn.00194.2020 PMC7474459

[tjp15192-bib-0002] Alexander, S. , Mathie, A. , & Peters, J. (2011). Ligand‐gated ion channels. British Journal of Pharmacology, 164, S115‐S135.

[tjp15192-bib-0003] Alvarez, F. J. , Pearson, J. C. , Harrington, D. , Dewey, D. , Torbeck, L. , & Fyffe, R. E. (1998). Distribution of 5‐hydroxytryptamine‐immunoreactive boutons on alpha‐motoneurons in the lumbar spinal cord of adult cats. Journal of Comparative Neurology, 393(1), 69–83. 9520102

[tjp15192-bib-0004] Arber, S. , & Costa, R. M. (2022). Networking brainstem and basal ganglia circuits for movement. Nature Reviews. Neuroscience, 23(6), 342–360. 35422525 10.1038/s41583-022-00581-w

[tjp15192-bib-0005] Balaban, C. D. (2002). Neural substrates linking balance control and anxiety. Physiology & Behavior, 77(4–5), 469–475. 12526985 10.1016/s0031-9384(02)00935-6

[tjp15192-bib-0006] Barnes, N. M. , & Sharp, T. (1999). A review of central 5‐HT receptors and their function. Neuropharmacology, 38(8), 1083–1152. 10462127 10.1016/s0028-3908(99)00010-6

[tjp15192-bib-0007] Batsikadze, G. , Paulus, W. , Kuo, M. F. , & Nitsche, M. A. (2013). Effect of serotonin on paired associative stimulation‐induced plasticity in the human motor cortex. Neuropsychopharmacology, 38(11), 2260–2267. 23680943 10.1038/npp.2013.127 PMC3773677

[tjp15192-bib-0008] Bayliss, D. A. , Umemiya, M. , & Berger, A. J. (1995). Inhibition of N‐ and P‐type calcium currents and the after‐hyperpolarization in rat motoneurones by serotonin. Journal of Physiology, 485(3), 635–647. 7562606 10.1113/jphysiol.1995.sp020758 PMC1158033

[tjp15192-bib-0009] Belcher, G. , Ryall, R. W. , & Schaffner, R. (1978). The differential effects of 5‐hydroxytryptamine, noradrenaline and raphe stimulation on nociceptive and non‐nociceptive dorsal horn interneurones in the cat. Brain Research, 151(2), 307–321. 679011 10.1016/0006-8993(78)90887-9

[tjp15192-bib-0010] Benarroch, E. E. (2018). Locus coeruleus. Cell and Tissue Research, 373(1), 221–232. 28687925 10.1007/s00441-017-2649-1

[tjp15192-bib-0011] Bennett, D. J. , Hultborn, H. , Fedirchuk, B. , & Gorassini, M. (1998). Synaptic activation of plateaus in hindlimb motoneurons of decerebrate cats. Journal of Neurophysiology, 80(4), 2023–2037. 9772258 10.1152/jn.1998.80.4.2023

[tjp15192-bib-0012] Berger, A. J. , & Takahashi, T. (1990). Serotonin enhances a low‐voltage‐activated calcium current in rat spinal motoneurons. Journal of Neuroscience, 10(6), 1922–1928. 2355258 10.1523/JNEUROSCI.10-06-01922.1990 PMC6570293

[tjp15192-bib-0013] Bos, R. , Drouillas, B. , Bouhadfane, M. , Pecchi, E. , Trouplin, V. , Korogod, S. M. , & Brocard, F. (2021). Trpm5 channels encode bistability of spinal motoneurons and ensure motor control of hindlimbs in mice. Nature Communications, 12(1), 6815. 10.1038/s41467-021-27113-x PMC8613399 34819493

[tjp15192-bib-0014] Bos, R. , Harris‐Warrick, R. M. , Brocard, C. , Demianenko, L. E. , Manuel, M. , Zytnicki, D. , Korogod, S. M. , & Brocard, F. (2018). Kv1.2 channels promote nonlinear spiking motoneurons for powering up locomotion. Cell Reports, 22(12), 3315–3327. 29562186 10.1016/j.celrep.2018.02.093 PMC5907934

[tjp15192-bib-0015] Celada, P. , Puig, M. V. , & Artigas, F. (2013). Serotonin modulation of cortical neurons and networks. Frontiers in Integrative Neuroscience, 7, 25. 23626526 10.3389/fnint.2013.00025 PMC3630391

[tjp15192-bib-0016] Cespuglio, R. , Faradji, H. , Gomez, M. E. , & Jouvet, M. (1981). Single unit recordings in the nuclei raphe dorsalis and magnus during the sleep‐waking cycle of semi‐chronic prepared cats. Neuroscience Letters, 24(2), 133–138. 7254710 10.1016/0304-3940(81)90236-6

[tjp15192-bib-0017] Classen, J. , Liepert, J. , Wise, S. P. , Hallett, M. , & Cohen, L. G. (1998). Rapid plasticity of human cortical movement representation induced by practice. Journal of Neurophysiology, 79(2), 1117–1123. 9463469 10.1152/jn.1998.79.2.1117

[tjp15192-bib-0018] Cotel, F. , Exley, R. , Cragg, S. J. , & Perrier, J. F. (2013). Serotonin spillover onto the axon initial segment of motoneurons induces central fatigue by inhibiting action potential initiation. Proceedings of the National Academy of Sciences, USA, 110(12), 4774–4779. 10.1073/pnas.1216150110 PMC3607056 23487756

[tjp15192-bib-0019] D'Amico, J. M. , Butler, A. A. , Heroux, M. E. , Cotel, F. , Perrier, J. M. , Butler, J. E. , Gandevia, S. C. , & Taylor, J. L. (2017). Human motoneurone excitability is depressed by activation of serotonin 1A receptors with buspirone. Journal of Physiology, 595(5), 1763–1773. 27859267 10.1113/JP273200 PMC5330870

[tjp15192-bib-0020] D'Amico, J. M. , Condliffe, E. G. , Martins, K. J. , Bennett, D. J. , & Gorassini, M. A. (2014). Recovery of neuronal and network excitability after spinal cord injury and implications for spasticity. Frontiers in Integrative Neuroscience, 8, 36. 24860447 10.3389/fnint.2014.00036 PMC4026713

[tjp15192-bib-0021] D'Amico, J. M. , Murray, K. C. , Li, Y. , Chan, K. M. , Finlay, M. G. , Bennett, D. J. , & Gorassini, M. A. (2013). Constitutively active 5‐HT2/alpha1 receptors facilitate muscle spasms after human spinal cord injury. Journal of Neurophysiology, 109(6), 1473–1484. 23221402 10.1152/jn.00821.2012 PMC3602940

[tjp15192-bib-0022] Deardorff, A. S. , Romer, S. H. , & Fyffe, R. E. W. (2021). Location, location, location: The organization and roles of potassium channels in mammalian motoneurons. Journal of Physiology, 599(5), 1391–1420. 33449375 10.1113/JP278675 PMC12993820

[tjp15192-bib-0023] Deffains, M. , & Bergman, H. (2015). Striatal cholinergic interneurons and cortico‐striatal synaptic plasticity in health and disease. Movement Disorders, 30(8), 1014–1025. 26095280 10.1002/mds.26300

[tjp15192-bib-0024] Delgado‐Lezama, R. , Perrier, J. F. , Nedergaard, S. , Svirskis, G. , & Hounsgaard, J. (1997). Metabotropic synaptic regulation of intrinsic response properties of turtle spinal motoneurones. Journal of Physiology, 504(1), 97–102. 9350621 10.1111/j.1469-7793.1997.097bf.x PMC1159939

[tjp15192-bib-0025] Eccles, J. C. , & McGeer, P. L. (1979). Ionotropic and metabotropic neurotransmission. Trends in Neuroscience, 2, 39–40.

[tjp15192-bib-0026] Elliott, P. , & Wallis, D. I. (1992). Serotonin and L‐norepinephrine as mediators of altered excitability in neonatal rat motoneurons studied in vitro. Neuroscience, 47(3), 533–544. 1584408 10.1016/0306-4522(92)90163-v

[tjp15192-bib-0027] Fedirchuk, B. , & Dai, Y. (2004). Monoamines increase the excitability of spinal neurones in the neonatal rat by hyperpolarizing the threshold for action potential production. Journal of Physiology, 557(2), 355–361. 15090607 10.1113/jphysiol.2004.064022 PMC1665108

[tjp15192-bib-0028] Foley, R. C. A. , & Kalmar, J. M. (2019). Estimates of persistent inward current in human motor neurons during postural sway. Journal of Neurophysiology, 122(5), 2095–2110. 31533012 10.1152/jn.00254.2019 PMC6879958

[tjp15192-bib-0029] Fornal, C. A. , Martin‐Cora, F. J. , & Jacobs, B. L. (2006). “Fatigue” of medullary but not mesencephalic raphe serotonergic neurons during locomotion in cats. Brain Research, 1072(1), 55–61. 16412992 10.1016/j.brainres.2005.12.007

[tjp15192-bib-0030] Fung, S. I. , Chan, J. Y. , Manzoni, D. , White, S. R. , Lai, Y. Y. , Strahlendorf, H. K. , Zhuo, H. , Liu, R. H. , Reddy, V. K. , & Barnes, C. D. (1994). Cotransmitter‐mediated locus coeruleus action on motoneurons. Brain Research Bulletin, 35(5–6), 423–432. 7859099 10.1016/0361-9230(94)90155-4

[tjp15192-bib-0031] Gerdelat‐Mas, A. , Loubinoux, I. , Tombari, D. , Rascol, O. , Chollet, F. , & Simonetta‐Moreau, M. (2005). Chronic administration of selective serotonin reuptake inhibitor (SSRI) paroxetine modulates human motor cortex excitability in healthy subjects. Neuroimage, 27(2), 314–322. 16019236 10.1016/j.neuroimage.2005.05.009

[tjp15192-bib-0032] Goodlich, B. I. , Horan, S. A. , & Kavanagh, J. J. (2022). Blockade of 5‐HT2 receptors suppresses rate of torque development and motor unit discharge rate during rapid contractions. Journal of Neurophysiology, 127(1), 150–160. 34936830 10.1152/jn.00470.2021

[tjp15192-bib-0033] Gorassini, M. , Yang, J. F. , Siu, M. , & Bennett, D. J. (2002). Intrinsic activation of human motoneurons: Possible contribution to motor unit excitation. Journal of Neurophysiology, 87(4), 1850–1858. 11929906 10.1152/jn.00024.2001

[tjp15192-bib-0034] Grunnet, M. , Jespersen, T. , & Perrier, J. F. (2004). 5‐HT1A receptors modulate small‐conductance Ca2+‐activated K+ channels. Journal of Neuroscience Research, 78(6), 845–854. 15521063 10.1002/jnr.20318

[tjp15192-bib-0035] Hachoumi, L. , Rensner, R. , Richmond, C. , Picton, L. , Zhang, H. , & Sillar, K. T. (2022). Bimodal modulation of short‐term motor memory via dynamic sodium pumps in a vertebrate spinal cord. Current Biology, 32, 1038–1048.e1032. 35104440 10.1016/j.cub.2022.01.012 PMC9616794

[tjp15192-bib-0036] Hassan, A. , Thompson, C. K. , Negro, F. , Cummings, M. , Powers, R. K. , Heckman, C. J. , Dewald, J. P. A. , & McPherson, L. M. (2020). Impact of parameter selection on estimates of motoneuron excitability using paired motor unit analysis. Journal of Neural Engineering, 17(1), 016063. 31801123 10.1088/1741-2552/ab5eda PMC7295184

[tjp15192-bib-0037] Hassan, A. S. , Fajardo, M. E. , Cummings, M. , McPherson, L. M. , Negro, F. , Dewald, J. P. A. , Heckman, C. J. , & Pearcey, G. E. (2021). Estimates of persistent inward currents are reduced in upper limb motor units of older adults. Journal of Physiology, 599(21), 4865–4882. 34505294 10.1113/JP282063 PMC8560565

[tjp15192-bib-0038] Heckman, C. J. , & Binder, M. D. (1991). Analysis of Ia‐inhibitory synaptic input to cat spinal motoneurons evoked by vibration of antagonist muscles. Journal of Neurophysiology, 66(6), 1888–1893. 1812223 10.1152/jn.1991.66.6.1888

[tjp15192-bib-0039] Heckman, C. J. , Hyngstrom, A. S. , & Johnson, M. D. (2008). Active properties of motoneurone dendrites: Diffuse descending neuromodulation, focused local inhibition. Journal of Physiology, 586(5), 1225–1231. 17947305 10.1113/jphysiol.2007.145078 PMC2375668

[tjp15192-bib-0040] Heckman, C. J. , Johnson, M. , Mottram, C. , & Schuster, J. (2008). Persistent inward currents in spinal motoneurons and their influence on human motoneuron firing patterns. The Neuroscientist, 14(3), 264–275. 18381974 10.1177/1073858408314986 PMC3326417

[tjp15192-bib-0041] Heckmann, C. J. , Gorassini, M. A. , & Bennett, D. J. (2005). Persistent inward currents in motoneuron dendrites: Implications for motor output. Muscle & Nerve, 31(2), 135–156. 15736297 10.1002/mus.20261

[tjp15192-bib-0042] Henderson, T. T. , Thorstensen, J. R. , Morrison, S. , Tucker, M. G. , & Kavanagh, J. J. (2022). Physiological tremor is suppressed and force steadiness is enhanced with increased availability of serotonin regardless of muscle fatigue. Journal of Neurophysiology, 127(1), 27–37. 34851768 10.1152/jn.00403.2021

[tjp15192-bib-0043] Herbert, R. D. , & Gandevia, S. C. (1999). Twitch interpolation in human muscles: Mechanisms and implications for measurement of voluntary activation. Journal of Neurophysiology, 82(5), 2271–2283. 10561405 10.1152/jn.1999.82.5.2271

[tjp15192-bib-0044] Hery, F. , Faudon, M. , & Ternaux, J. P. (1982). In vivo release of serotonin in two raphe nuclei (raphe dorsalis and magnus) of the cat. Brain Research Bulletin, 8(2), 123–129. 7066704 10.1016/0361-9230(82)90038-7

[tjp15192-bib-0045] Hornung, J. P. (2003). The human raphe nuclei and the serotonergic system. Journal of Chemical Neuroanatomy, 26(4), 331–343. 14729135 10.1016/j.jchemneu.2003.10.002

[tjp15192-bib-0046] Hounsgaard, J. , Hultborn, H. , Jespersen, B. , & Kiehn, O. (1988). Bistability of alpha‐motoneurones in the decerebrate cat and in the acute spinal cat after intravenous 5‐hydroxytryptophan. Journal of Physiology, 405(1), 345–367. 3267153 10.1113/jphysiol.1988.sp017336 PMC1190979

[tjp15192-bib-0047] Hounsgaard, J. , & Kiehn, O. (1989). Serotonin‐induced bistability of turtle motoneurones caused by a nifedipine‐sensitive calcium plateau potential. Journal of Physiology, 414(1), 265–282. 2607432 10.1113/jphysiol.1989.sp017687 PMC1189141

[tjp15192-bib-0048] Hoyer, D. , & Martin, G. (1997). 5‐HT receptor classification and nomenclature: Towards a harmonization with the human genome. Neuropharmacology, 36(4–5), 419–428. 9225265 10.1016/s0028-3908(97)00036-1

[tjp15192-bib-0049] Hsiao, C. F. , Trueblood, P. R. , Levine, M. S. , & Chandler, S. H. (1997). Multiple effects of serotonin on membrane properties of trigeminal motoneurons in vitro. Journal of Neurophysiology, 77(6), 2910–2924. 9212246 10.1152/jn.1997.77.6.2910

[tjp15192-bib-0050] Huh, S. , Siripuram, R. , Lee, R. H. , Turkin, V. V. , O'Neill, D. , Hamm, T. M. , Heckman, C. J. , & Manuel, M. (2017). PICs in motoneurons do not scale with the size of the animal: A possible mechanism for faster speed of muscle contraction in smaller species. Journal of Neurophysiology, 118(1), 93–102. 28356469 10.1152/jn.00045.2017 PMC5494365

[tjp15192-bib-0051] Hultborn, H. , Denton, M. E. , Wienecke, J. , & Nielsen, J. B. (2003). Variable amplification of synaptic input to cat spinal motoneurones by dendritic persistent inward current. Journal of Physiology, 552(3), 945–952. 14500771 10.1113/jphysiol.2003.050971 PMC2343455

[tjp15192-bib-0052] Hyngstrom, A. , Johnson, M. , Schuster, J. , & Heckman, C. J. (2008). Movement‐related receptive fields of spinal motoneurones with active dendrites. Journal of Physiology, 586(6), 1581–1593. 18238818 10.1113/jphysiol.2007.149146 PMC2375694

[tjp15192-bib-0053] Hyngstrom, A. S. , Johnson, M. D. , Miller, J. F. , & Heckman, C. J. (2007). Intrinsic electrical properties of spinal motoneurons vary with joint angle. Nature Neuroscience, 10(3), 363–369. 17293858 10.1038/nn1852

[tjp15192-bib-0054] Ikeda, H. , Adachi, K. , Fujita, S. , Tomiyama, K. , Saigusa, T. , Kobayashi, M. , Koshikawa, N. , & Waddington, J. L. (2015). Investigating complex basal ganglia circuitry in the regulation of motor behaviour, with particular focus on orofacial movement. Behavioural Pharmacology, 26(1 and 2), 18–32. 25485640 10.1097/FBP.0000000000000118

[tjp15192-bib-0055] Ilic, T. V. , Korchounov, A. , & Ziemann, U. (2002). Complex modulation of human motor cortex excitability by the specific serotonin re‐uptake inhibitor sertraline. Neuroscience Letters, 319(2), 116–120. 11825684 10.1016/s0304-3940(01)02563-0

[tjp15192-bib-0056] Imai, H. , Steindler, D. A. , & Kitai, S. T. (1986). The organization of divergent axonal projections from the midbrain raphe nuclei in the rat. Journal of Comparative Neurology, 243(3), 363–380. 2419370 10.1002/cne.902430307

[tjp15192-bib-0057] Jackson, D. A. , & White, S. R. (1990). Receptor subtypes mediating facilitation by serotonin of excitability of spinal motoneurons. Neuropharmacology, 29(9), 787–797. 2149874 10.1016/0028-3908(90)90151-g

[tjp15192-bib-0058] Jacobs, B. L. , & Fornal, C. A. (1997). Serotonin and motor activity. Current Opinion in Neurobiology, 7(6), 820–825. 9464975 10.1016/s0959-4388(97)80141-9

[tjp15192-bib-0059] Jacobs, B. L. , Martin‐Cora, F. J. , & Fornal, C. A. (2002). Activity of medullary serotonergic neurons in freely moving animals. Brain Research. Brain Research Reviews, 40(1–3), 45–52. 12589905 10.1016/s0165-0173(02)00187-x

[tjp15192-bib-0060] Jankowska, E. , Hammar, I. , Chojnicka, B. , & Heden, C. H. (2000). Effects of monoamines on interneurons in four spinal reflex pathways from group I and/or group II muscle afferents. European Journal of Neuroscience, 12(2), 701–714. 10712650 10.1046/j.1460-9568.2000.00955.x

[tjp15192-bib-0061] Jankowska, E. , Riddell, J. S. , Skoog, B. , & Noga, B. R. (1993). Gating of transmission to motoneurones by stimuli applied in the locus coeruleus and raphe nuclei of the cat. Journal of Physiology, 461(1), 705–722. 8394430 10.1113/jphysiol.1993.sp019537 PMC1175281

[tjp15192-bib-0062] Jones, B. E. (2008). Modulation of cortical activation and behavioral arousal by cholinergic and orexinergic systems. Annals of the New York Academy of Sciences, 1129(1), 26–34. 18591466 10.1196/annals.1417.026

[tjp15192-bib-0063] Jordan, L. M. , Kenshalo, D. R., Jr. , Martin, R. F. , Haber, L. H. , & Willis, W. D. (1979). Two populations of spinothalamic tract neurons with opposite responses to 5‐hydroxytryptamine. Brain Research, 164(1–2), 342–346. 218688 10.1016/0006-8993(79)90034-9

[tjp15192-bib-0064] Joyce, J. N. , Shane, A. , Lexow, N. , Winokur, A. , Casanova, M. F. , & Kleinman, J. E. (1993). Serotonin uptake sites and serotonin receptors are altered in the limbic system of schizophrenics. Neuropsychopharmacology, 8(4), 315–336. 8512620 10.1038/npp.1993.32

[tjp15192-bib-0065] Kavanagh, J. J. , McFarland, A. J. , & Taylor, J. L. (2019). Enhanced availability of serotonin increases activation of unfatigued muscle but exacerbates central fatigue during prolonged sustained contractions. Journal of Physiology, 597(1), 319–332. 30328105 10.1113/JP277148 PMC6312415

[tjp15192-bib-0066] Kawashima, T. (2018). The role of the serotonergic system in motor control. Neuroscience Research, 129, 32–39. 28774814 10.1016/j.neures.2017.07.005

[tjp15192-bib-0067] Kernell, D. (2006). The motoneurone and its muscle fibres. Oxford University Press, Oxford, New York.

[tjp15192-bib-0068] Klass, M. , Duchateau, J. , Rabec, S. , Meeusen, R. , & Roelands, B. (2016). Noradrenaline reuptake inhibition impairs cortical output and limits endurance time. Medicine and Science in Sports and Exercise, 48(6), 1014–1023. 26784275 10.1249/MSS.0000000000000879

[tjp15192-bib-0069] Klass, M. , Roelands, B. , Levenez, M. , Fontenelle, V. , Pattyn, N. , Meeusen, R. , & Duchateau, J. (2012). Effects of noradrenaline and dopamine on supraspinal fatigue in well‐trained men. Medicine and Science in Sports and Exercise, 44(12), 2299–2308. 22776872 10.1249/MSS.0b013e318265f356

[tjp15192-bib-0070] Klaus, A. , Alves da Silva, J. , & Costa, R. M. (2019). What, if, and when to move: Basal ganglia circuits and self‐paced action initiation. Annual Review of Neuroscience, 42(1), 459–483. 10.1146/annurev-neuro-072116-031033 31018098

[tjp15192-bib-0071] Kuo, J. J. , Lee, R. H. , Johnson, M. D. , Heckman, H. M. , & Heckman, C. J. (2003). Active dendritic integration of inhibitory synaptic inputs in vivo. Journal of Neurophysiology, 90(6), 3617–3624. 12944534 10.1152/jn.00521.2003

[tjp15192-bib-0072] Lee, R. H. , & Heckman, C. J. (1996). Influence of voltage‐sensitive dendritic conductances on bistable firing and effective synaptic current in cat spinal motoneurons in vivo. Journal of Neurophysiology, 76(3), 2107–2110. 8890322 10.1152/jn.1996.76.3.2107

[tjp15192-bib-0073] Lee, R. H. , & Heckman, C. J. (1998). Bistability in spinal motoneurons in vivo: Systematic variations in persistent inward currents. Journal of Neurophysiology, 80(2), 583–593. 9705452 10.1152/jn.1998.80.2.583

[tjp15192-bib-0074] Lee, R. H. , & Heckman, C. J. (2000). Adjustable amplification of synaptic input in the dendrites of spinal motoneurons in vivo. Journal of Neuroscience, 20(17), 6734–6740. 10964980 10.1523/JNEUROSCI.20-17-06734.2000 PMC6772971

[tjp15192-bib-0075] Loubinoux, I. , Boulanouar, K. , Ranjeva, J. P. , Carel, C. , Berry, I. , Rascol, O. , Celsis, P. , & Chollet, F. (1999). Cerebral functional magnetic resonance imaging activation modulated by a single dose of the monoamine neurotransmission enhancers fluoxetine and fenozolone during hand sensorimotor tasks. Journal of Cerebral Blood Flow and Metabolism, 19(12), 1365–1375. 10598941 10.1097/00004647-199912000-00010

[tjp15192-bib-0076] Loubinoux, I. , Pariente, J. , Rascol, O. , Celsis, P. , & Chollet, F. (2002). Selective serotonin reuptake inhibitor paroxetine modulates motor behavior through practice. A double‐blind, placebo‐controlled, multi‐dose study in healthy subjects. Neuropsychologia, 40(11), 1815–1821. 12062893 10.1016/s0028-3932(02)00030-1

[tjp15192-bib-0077] Lucas, J. J. , & Hen, R. (1995). New players in the 5‐HT receptor field: Genes and knockouts. Trends in Pharmacological Sciences, 16(7), 246–252. 7667899 10.1016/s0165-6147(00)89034-3

[tjp15192-bib-0078] Marvin, G. , Sharma, A. , Aston, W. , Field, C. , Kendall, M. J. , & Jones, D. A. (1997). The effects of buspirone on perceived exertion and time to fatigue in man. Experimental Physiology, 82(6), 1057–1060. 9413736 10.1113/expphysiol.1997.sp004080

[tjp15192-bib-0079] McDonnell, M. N. , Zipser, C. , Darmani, G. , Ziemann, U. , & Muller‐Dahlhaus, F. (2018). The effects of a single dose of fluoxetine on practice‐dependent plasticity. Clinical Neurophysiology, 129(7), 1349–1356. 29729588 10.1016/j.clinph.2018.04.604

[tjp15192-bib-0080] McGinty, D. J. , & Harper, R. M. (1976). Dorsal raphe neurons: Depression of firing during sleep in cats. Brain Research, 101(3), 569–575. 1244990 10.1016/0006-8993(76)90480-7

[tjp15192-bib-0081] Meeusen, R. , Piacentini, M. F. , Van Den Eynde, S. , Magnus, L. , & De Meirleir, K. (2001). Exercise performance is not influenced by a 5‐HT reuptake inhibitor. International Journal of Sports Medicine, 22(05), 329–336. 11510868 10.1055/s-2001-15648

[tjp15192-bib-0082] Mesquita, R. N. O. , Taylor, J. L. , Trajano, G. S. , Skarabot, J. , Holobar, A. , Goncalves, B. A. M. , & Blazevich, A. J. (2022). Effects of reciprocal inhibition and whole‐body relaxation on persistent inward currents estimated by two different methods. Journal of Physiology, 600(11), 2765–2787. 35436349 10.1113/JP282765 PMC9325475

[tjp15192-bib-0083] Mille, T. , Quilgars, C. , Cazalets, J. R. , & Bertrand, S. S. (2021). Acetylcholine and spinal locomotor networks: The insider. Physiological Reports, 9(3), e14736. 33527727 10.14814/phy2.14736 PMC7851432

[tjp15192-bib-0084] Murray, K. C. , Stephens, M. J. , Ballou, E. W. , Heckman, C. J. , & Bennett, D. J. (2011). Motoneuron excitability and muscle spasms are regulated by 5‐HT2B and 5‐HT2C receptor activity. Journal of Neurophysiology, 105(2), 731–748. 20980537 10.1152/jn.00774.2010 PMC3059173

[tjp15192-bib-0085] Naicker, P. , Anoopkumar‐Dukie, S. , Grant, G. D. , & Kavanagh, J. J. (2017). Anticholinergic activity in the nervous system: Consequences for visuomotor function. Physiology & Behavior, 170, 6–11. 27965143 10.1016/j.physbeh.2016.12.010

[tjp15192-bib-0086] Newsholme, E. A. , Acworth, I. N. , & Blomstrand, E. (1987). Amino acids, brain neurotransmitters and a functional link between muscle and brain that is important in sustained exercise. In G. Benzi (Ed.). Advances in myochemistry (pp. 127–133). London : John Libbey.

[tjp15192-bib-0087] Newsholme, E. A. , & Blomstrand, E. (2006). Branched‐chain amino acids and central fatigue. Journal of Nutrition, 136(1), 274S‐276S. 16365097 10.1093/jn/136.1.274S

[tjp15192-bib-0088] Nichols, D. E. , & Nichols, C. D. (2008). Serotonin receptors. Chemical Reviews, 108(5), 1614–1641. 18476671 10.1021/cr078224o

[tjp15192-bib-0089] Nitsche, M. A. , Kuo, M. F. , Karrasch, R. , Wachter, B. , Liebetanz, D. , & Paulus, W. (2009). Serotonin affects transcranial direct current‐induced neuroplasticity in humans. Biological Psychiatry, 66(5), 503–508. 19427633 10.1016/j.biopsych.2009.03.022

[tjp15192-bib-0090] Noga, B. R. , Turkson, R. P. , Xie, S. , Taberner, A. , Pinzon, A. , & Hentall, I. D. (2017). Monoamine release in the cat lumbar spinal cord during fictive locomotion evoked by the mesencephalic locomotor region. Frontiers in Neural Circuits, 11, 59. 28912689 10.3389/fncir.2017.00059 PMC5582069

[tjp15192-bib-0091] Orssatto, L. B. R. , Borg, D. N. , Blazevich, A. J. , Sakugawa, R. L. , Shield, A. J. , & Trajano, G. S. (2021). Intrinsic motoneuron excitability is reduced in soleus and tibialis anterior of older adults. Geroscience, 43(6), 2719–2735. 34716899 10.1007/s11357-021-00478-z PMC8556797

[tjp15192-bib-0092] Orssatto, L. B. R. , Mackay, K. , Shield, A. J. , Sakugawa, R. L. , Blazevich, A. J. , & Trajano, G. S. (2021). Estimates of persistent inward currents increase with the level of voluntary drive in low‐threshold motor units of plantar flexor muscles. Journal of Neurophysiology, 125(5), 1746–1754. 33788617 10.1152/jn.00697.2020

[tjp15192-bib-0093] Pavlos, N. J. , & Friedman, P. A. (2017). GPCR signaling and trafficking: The long and short of it. Trends in Endocrinology and Metabolism, 28(3), 213–226. 27889227 10.1016/j.tem.2016.10.007 PMC5326587

[tjp15192-bib-0094] Pazos, A. , Cortes, R. , & Palacios, J. M. (1985). Quantitative autoradiographic mapping of serotonin receptors in the rat brain. II. Serotonin‐2 receptors. Brain Research, 346(2), 231–249. 4052777 10.1016/0006-8993(85)90857-1

[tjp15192-bib-0095] Pazos, A. , & Palacios, J. M. (1985). Quantitative autoradiographic mapping of serotonin receptors in the rat brain. I. Serotonin‐1 receptors. Brain Research, 346(2), 205–230. 4052776 10.1016/0006-8993(85)90856-x

[tjp15192-bib-0096] Pearcey, G. E. P. , Khurram, O. U. , Beauchamp, J. A. , & Heckman, C. J. (2020). Exploring the effects of Ia reciprocal inhibition on neuromodulatory commands in the human lower limb. Journal of the Federation of of American Societies for Experimental Biology, 34, 1–1.

[tjp15192-bib-0097] Peroutka, S. J. (1990). 5‐hydroxytryptamine receptor subtypes. Pharmacology & Toxicology, 67, 373–383. 2096381 10.1111/j.1600-0773.1990.tb00848.x

[tjp15192-bib-0098] Perrier, J. F. , Alaburda, A. , & Hounsgaard, J. (2003). 5‐HT1A receptors increase excitability of spinal motoneurons by inhibiting a TASK‐1‐like K+ current in the adult turtle. Journal of Physiology, 548(2), 485–492. 12626670 10.1113/jphysiol.2002.037952 PMC2342869

[tjp15192-bib-0099] Perrier, J. F. , & Cotel, F. (2008a). Serotonin differentially modulates the intrinsic properties of spinal motoneurons from the adult turtle. Journal of Physiology, 586(5), 1233–1238. 18096602 10.1113/jphysiol.2007.145706 PMC2375675

[tjp15192-bib-0100] Perrier, J. F. , & Cotel, F. (2008b). Serotonin differentially modulates the intrinsic properties of spinal motoneurons from the adult turtle. Journal of Physiology, 586(5), 1233–1238. 18096602 10.1113/jphysiol.2007.145706 PMC2375675

[tjp15192-bib-0101] Perrier, J. F. , & Cotel, F. (2015). Serotonergic modulation of spinal motor control. Current Opinion in Neurobiology, 33, 1–7. 25553359 10.1016/j.conb.2014.12.008

[tjp15192-bib-0102] Perrier, J. F. , & Delgado‐Lezama, R. (2005). Synaptic release of serotonin induced by stimulation of the raphe nucleus promotes plateau potentials in spinal motoneurons of the adult turtle. Journal of Neuroscience, 25(35), 7993–7999. 16135756 10.1523/JNEUROSCI.1957-05.2005 PMC6725458

[tjp15192-bib-0103] Perrier, J. F. , & Hounsgaard, J. (2003). 5‐HT2 receptors promote plateau potentials in turtle spinal motoneurons by facilitating an L‐type calcium current. Journal of Neurophysiology, 89(2), 954–959. 12574471 10.1152/jn.00753.2002

[tjp15192-bib-0104] Perrier, J. F. , Rasmussen, H. B. , Christensen, R. K. , & Petersen, A. V. (2013). Modulation of the intrinsic properties of motoneurons by serotonin. Current Pharmaceutical Design, 19(24), 4371–4384. 23360270 10.2174/13816128113199990341

[tjp15192-bib-0105] Perrier, J. F. , Rasmussen, H. B. , Jorgensen, L. K. , & Berg, R. W. (2017). Intense activity of the raphe spinal pathway depresses motor activity via a serotonin dependent mhohechanism. Frontiers in Neural Circuits, 11, 111. 29375322 10.3389/fncir.2017.00111 PMC5767281

[tjp15192-bib-0106] Phillis, J. W. , Tebecis, A. K. , & York, D. H. (1968). Depression of spinal motoneurones by noradrenaline, 5‐hydroxytryptamine and histamine. European Journal of Pharmacology, 4(4), 471–475. 4302133 10.1016/0014-2999(68)90037-x

[tjp15192-bib-0107] Picton, L. D. , & Sillar, K. T. (2016). Mechanisms underlying the endogenous dopaminergic inhibition of spinal locomotor circuit function in Xenopus tadpoles. Scientific Reports, 6(1), 35749. 27760989 10.1038/srep35749 PMC5071771

[tjp15192-bib-0108] Pleger, B. , Schwenkreis, P. , Grunberg, C. , Malin, J. P. , & Tegenthoff, M. (2004). Fluoxetine facilitates use‐dependent excitability of human primary motor cortex. Clinical Neurophysiology, 115(9), 2157–2163. 15294219 10.1016/j.clinph.2004.04.015

[tjp15192-bib-0109] Pollak Dorocic, I. , Furth, D. , Xuan, Y. , Johansson, Y. , Pozzi, L. , Silberberg, G. , Carlen, M. , & Meletis, K. (2014). A whole‐brain atlas of inputs to serotonergic neurons of the dorsal and median raphe nuclei. Neuron, 83(3), 663–678. 25102561 10.1016/j.neuron.2014.07.002

[tjp15192-bib-0110] Powers, R. K. , Turker, K. S. , & Binder, M. D. (2002). What can be learned about motoneurone properties from studying firing patterns? Advances in Experimental Medicine and Biology, 508, 199–205. 12171112 10.1007/978-1-4615-0713-0_24

[tjp15192-bib-0111] Ridet, J. L. , Tamir, H. , & Privat, A. (1994). Direct immunocytochemical localization of 5‐hydroxytryptamine receptors in the adult rat spinal cord: A light and electron microscopic study using an anti‐idiotypic antiserum. Journal of Neuroscience Research, 38(1), 109–121. 8057387 10.1002/jnr.490380114

[tjp15192-bib-0112] Roelands, B. , Goekint, M. , Buyse, L. , Pauwels, F. , De Schutter, G. , Piacentini, F. , Hasegawa, H. , Watson, P. , & Meeusen, R. (2009). Time trial performance in normal and high ambient temperature: Is there a role for 5‐HT? European Journal of Applied Physiology, 107(1), 119–126. 19533165 10.1007/s00421-009-1109-3

[tjp15192-bib-0113] Schwindt, P. , & Crill, W. E. (1977). A persistent negative resistance in cat lumbar motoneurons. Brain Research, 120(1), 173–178. 832116 10.1016/0006-8993(77)90510-8

[tjp15192-bib-0114] Schwindt, P. C. , & Crill, W. E. (1980). Properties of a persistent inward current in normal and TEA‐injected motoneurons. Journal of Neurophysiology, 43(6), 1700–1724. 6251180 10.1152/jn.1980.43.6.1700

[tjp15192-bib-0115] Scullion, K. , Boychuk, J. A. , Yamakawa, G. R. , Rodych, J. T. G. , Nakanishi, S. T. , Seto, A. , Smith, V. M. , McCarthy, R. W. , Whelan, P. J. , Antle, M. C. , Pittman, Q. J. , & Teskey, G. C. (2013). Serotonin 1A receptors alter expression of movement representations. Journal of Neuroscience, 33(11), 4988–4999. 23486969 10.1523/JNEUROSCI.4241-12.2013 PMC6619014

[tjp15192-bib-0116] Sharples, S. A. , Koblinger, K. , Humphreys, J. M. , & Whelan, P. J. (2014). Dopamine: A parallel pathway for the modulation of spinal locomotor networks. Frontiers in Neural Circuits, 8, 55. 24982614 10.3389/fncir.2014.00055 PMC4059167

[tjp15192-bib-0117] Sogaard, K. , Gandevia, S. C. , Todd, G. , Petersen, N. T. , & Taylor, J. L. (2006). The effect of sustained low‐intensity contractions on supraspinal fatigue in human elbow flexor muscles. Journal of Physiology, 573(2), 511–523. 16556656 10.1113/jphysiol.2005.103598 PMC1779725

[tjp15192-bib-0118] Strachan, A. T. , Leiper, J. B. , & Maughan, R. J. (2004). Paroxetine administration failed to influence human exercise capacity, perceived effort or hormone responses during prolonged exercise in a warm environment. Experimental Physiology, 89(6), 657–664. 15328306 10.1113/expphysiol.2004.027839

[tjp15192-bib-0119] Strüder, H. K. , Hollmann, W. , Platen, P. , Donike, M. , Gotzmann, A. , & Weber, K. (1998). Influence of paroxetine, branched‐chain amino acids and tyrosine on neuroendocrine system responses and fatigue in humans. Hormone and Metabolic Research, 30(04), 188–194. 9623632 10.1055/s-2007-978864

[tjp15192-bib-0120] Takahashi, T. , & Berger, A. J. (1990). Direct excitation of rat spinal motoneurones by serotonin. Journal of Physiology, 423(1), 63–76. 1696989 10.1113/jphysiol.1990.sp018011 PMC1189746

[tjp15192-bib-0121] Teixeira‐Coelho, F. , Uendeles‐Pinto, J. P. , Serafim, A. C. , Wanner, S. P. , de Matos Coelho, M. , & Soares, D. D. (2014). The paroxetine effect on exercise performance depends on the aerobic capacity of exercising individuals. Journal of Sports Science & Medicine, 13, 232–243. 24790474 PMC3990874

[tjp15192-bib-0122] Terry, N. , & Margolis, K. G. (2017). Serotonergic mechanisms regulating the GI tract: Experimental evidence and therapeutic relevance. Handbook of Experiemntal Pharmacology, 239, 319–342. 10.1007/164_2016_103 PMC5526216 28035530

[tjp15192-bib-0123] Thompson, A. J. , & Lummis, S. C. (2006). 5‐HT3 receptors. Current Pharmaceutical Design, 12(28), 3615–3630. 17073663 10.2174/138161206778522029 PMC2664614

[tjp15192-bib-0124] Thorstensen, J. R. , Taylor, J. L. , & Kavanagh, J. J. (2021). Human corticospinal‐motoneuronal output is reduced with 5‐HT2 receptor antagonism. Journal of Neurophysiology, 125(4), 1279–1288. 33596722 10.1152/jn.00698.2020

[tjp15192-bib-0125] Thorstensen, J. R. , Taylor, J. L. , Tucker, M. G. , & Kavanagh, J. J. (2020). Enhanced serotonin availability amplifies fatigue perception and modulates the TMS‐induced silent period during sustained low‐intensity elbow flexions. Journal of Physiology, 598(13), 2685–2701. 32243582 10.1113/JP279347

[tjp15192-bib-0126] Thorstensen, J. R. , Tucker, M. G. , & Kavanagh, J. J. (2018). Antagonism of the D2 dopamine receptor enhances tremor but reduces voluntary muscle activation in humans. Neuropharmacology, 141, 343–352. 30171987 10.1016/j.neuropharm.2018.08.029

[tjp15192-bib-0127] Todd, A. J. , & Millar, J. (1983). Receptive fields and responses to ionophoretically applied noradrenaline and 5‐hydroxytryptamine of units recorded in laminae I‐III of cat dorsal horn. Brain Research, 288(1–2), 159–167. 6661615 10.1016/0006-8993(83)90090-2

[tjp15192-bib-0128] Trajano, G. S. , Taylor, J. L. , Orssatto, L. B. R. , McNulty, C. R. , & Blazevich, A. J. (2020). Passive muscle stretching reduces estimates of persistent inward current strength in soleus motor units. Journal of Experimental Biology, 223(Pt 21), jeb229922. 32978317 10.1242/jeb.229922

[tjp15192-bib-0129] Udina, E. , D'Amico, J. , Bergquist, A. J. , & Gorassini, M. A. (2010). Amphetamine increases persistent inward currents in human motoneurons estimated from paired motor‐unit activity. Journal of Neurophysiology, 103(3), 1295–1303. 20053846 10.1152/jn.00734.2009 PMC2887628

[tjp15192-bib-0130] Veasey, S. C. , Fornal, C. A. , Metzler, C. W. , & Jacobs, B. L. (1995). Response of serotonergic caudal raphe neurons in relation to specific motor activities in freely moving cats. Journal of Neuroscience, 15(7), 5346–5359. 7623157 10.1523/JNEUROSCI.15-07-05346.1995 PMC6577863

[tjp15192-bib-0131] Vertes, R. P. (1991). A PHA‐L analysis of ascending projections of the dorsal raphe nucleus in the rat. Journal of Comparative Neurology, 313(4), 643–668. 1783685 10.1002/cne.903130409

[tjp15192-bib-0132] Vitrac, C. , & Benoit‐Marand, M. (2017). Monoaminergic modulation of motor cortex function. Frontiers in Neural Circuits, 11, 72. 29062274 10.3389/fncir.2017.00072 PMC5640772

[tjp15192-bib-0133] Wikstrom, M. , Hill, R. , Hellgren, J. , & Grillner, S. (1995). The action of 5‐HT on calcium‐dependent potassium channels and on the spinal locomotor network in lamprey is mediated by 5‐HT1A‐like receptors. Brain Research, 678(1–2), 191–199. 7620887 10.1016/0006-8993(95)00183-q

[tjp15192-bib-0134] Wilson, M. A. , & Molliver, M. E. (1991). The organization of serotonergic projections to cerebral cortex in primates: Regional distribution of axon terminals. Neuroscience, 44(3), 537–553. 1754051 10.1016/0306-4522(91)90076-z

[tjp15192-bib-0135] Wilson, W. M. , & Maughan, R. J. (1992). Evidence for a possible role of 5‐hydroxytryptamine in the genesis of fatigue in man: Administration of paroxetine, a 5‐HT re‐uptake inhibitor, reduces the capacity to perform prolonged exercise. Experimental Physiology, 77(6), 921–924. 1489548 10.1113/expphysiol.1992.sp003660

[tjp15192-bib-0136] Zhang, H. Y. , Picton, L. , Li, W. C. , & Sillar, K. T. (2015). Mechanisms underlying the activity‐dependent regulation of locomotor network performance by the Na pump. Scientific Reports, 5, 16188. 26541477 10.1038/srep16188 PMC4635428

[tjp15192-bib-0137] Zhang, H. Y. , & Sillar K. T. (2012). Short‐term memory of motor network performance via activity‐dependent potentiation of Na/K pump function. Current Biology, 22(6), 526–531. 22405867 10.1016/j.cub.2012.01.058

